# The use of multiparametric CMR to predict impaired exercise capacity in hypertrophic cardiomyopathy

**DOI:** 10.1186/1532-429X-11-S1-P15

**Published:** 2009-01-28

**Authors:** Andrew S Flett, Caroline J Coats, Brian A Mist, Giovanni Quarta, Ferdinando Pasquale, Perry M Elliott, James C Moon

**Affiliations:** The Heart Hospital, London, UK

**Keywords:** Diastolic Dysfunction, Late Gadolinium Enhancement, Exercise Capacity, Hypertrophic Cardiomyopathy, Atrial Pressure

## Objective

To understand the role of CMR measured parameters including late gadolinium enhancement (LGE) on exercise capacity in patients with hypertrophic cardiomyopathy (HCM).

## Background

Exercise intolerance in HCM is complex, depending on multiple factors including diastolic dysfunction, outflow tract obstruction, ischemia, left atrial pressure, hypertrophy and fibrosis. We sought to determine the relative contributions of 5 CMR measured variables on exercise capacity in HCM: function, mass, left atrial area, resting LVOT obstruction and LGE.

## Methods

135 consecutive patients with HCM (median age 46.5 years, 21% female, 67% Caucasian) underwent cardiopulmonary metabolic exercise testing (measuring percent predicted peak oxygen consumption (%pVO_2_) and contrast CMR (for function, volumes, mass, left atrial area, presence of rest LVOT obstruction and LGE). Two independent investigators blinded to results of the opposing dataset performed analysis of exercise and CMR data. The extent of LGE was categorised as normal/minimal, moderate or extensive (3 point scale, 0, 1 or 2). Univariate and multivariate analysis was used to assess correlations of %pVO_2_ with CMR derived variables.

## Results

**Figure 1:**
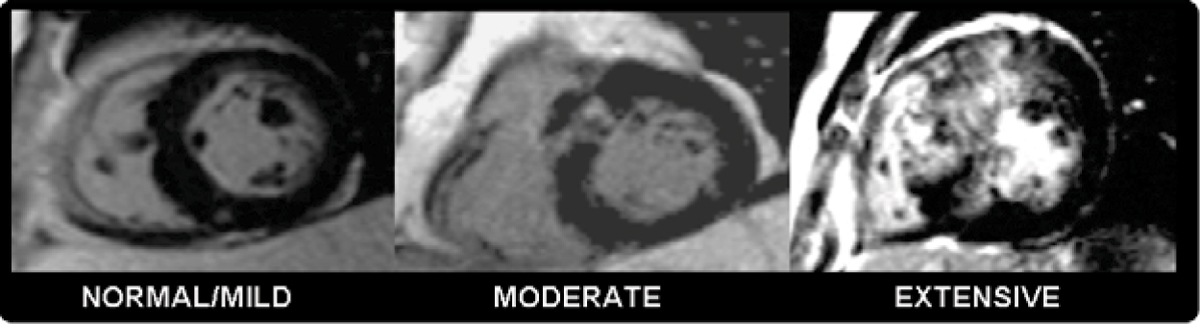
**The extent of late gadolinium enhancement assigned to 3 groups**.

**Figure 2:**
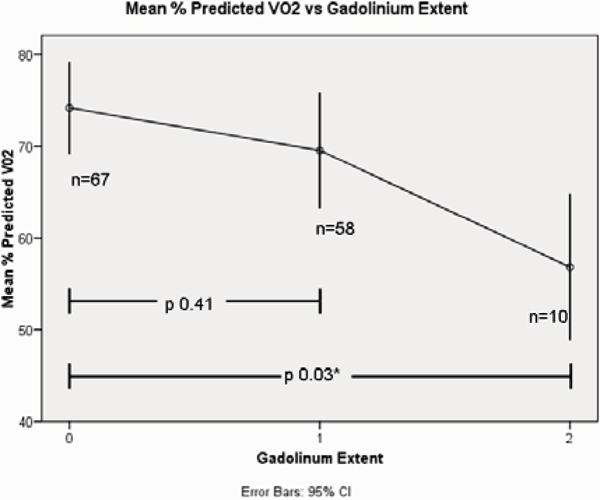
**Peak oxygen consumption falls with extent of LGE in patients with HCM**.

Normal/minimal, moderate and extensive LGE was present in 67 (50%), 58 (43%) and 10 (7%) respectively. 31(23%) had resting LVOT obstruction. On univariate analysis, %pVO_2_ was associated the presence of resting outflow tract obstruction (p = 0.03) and inversely asscociated with extensive LGE (p = 0.02). On multivariate analysis, the single significant factor associated with %pVO_2_ was LGE (p = 0.01). Although LGE and EF were associated (r = 0.232, p = 0.007), LGE was the independent predictor of %pVO_2_. If significant exercise impairment is defined as a %pVO_2_ <70%, moderate and extensive LGE independently predicts it with odds ratios of 2.2 (95% CI 1.0, 4.5 p = 0.04) and 18.9 (95% CI 1.9, 188.2, p = 0.01) respectively. Extensive LGE has low sensitivity (13%) but high specificity (98%) for predicting poor exercise capacity. Figures 1 and 2

## Conclusion

The main determinant of significant exercise limitation measured by multiparametric CMR in HCM is the presence of extensive LGE. Lesser amounts of LGE do not predict exercise capacity, highlighting the mulitfactorial nature of functional limitation in this condition.

